# Tumor Suppressor Function of the *SEMA3B* Gene in Human Lung and Renal Cancers

**DOI:** 10.1371/journal.pone.0123369

**Published:** 2015-05-11

**Authors:** Vitaly I. Loginov, Alexey A. Dmitriev, Vera N. Senchenko, Irina V. Pronina, Dmitry S. Khodyrev, Anna V. Kudryavtseva, George S. Krasnov, Ganna V. Gerashchenko, Larisa I. Chashchina, Tatiana P. Kazubskaya, Tatiana T. Kondratieva, Michael I. Lerman, Debora Angeloni, Eleonora A. Braga, Vladimir I. Kashuba

**Affiliations:** 1 Laboratory of Pathogenomics and Transcriptomics, Institute of General Pathology and Pathophysiology, Russian Academy of Medical Sciences, 125315, Moscow, Russia; 2 Laboratory of Molecular Genetics of Complex Inherited Diseases, Research Center of Medical Genetics, Russian Academy of Medical Sciences, 115478, Moscow, Russia; 3 Laboratory of Structural and Functional Genomics, Engelhardt Institute of Molecular Biology, Russian Academy of Sciences, 119991, Moscow, Russia; 4 Department of Pathomorphology, P.A. Herzen Moscow Cancer Research Institute, Ministry of Healthcare of the Russian Federation, 125284, Moscow, Russia; 5 Laboratory of Genetics, Federal Research Clinical Center of Federal Medical and Biological Agency of Russia, 115682, Moscow, Russia; 6 Laboratory of Biotechnology, Mechnikov Research Institute for Vaccines and Sera, Russian Academy of Medical Sciences, 105064, Moscow, Russia; 7 Department of Molecular Oncogenetics, Institute of Molecular Biology and Genetics, National Academy of Sciences of Ukraine, 03680, Kiev, Ukraine; 8 Research Institute of Clinical Oncology, N.N. Blokhin Cancer Research Center, Russian Academy of Medical Sciences, 115478, Moscow, Russia; 9 Scientific Board, Affina Biotechnologies, 06902, Stamford, CT, USA; 10 The Institute of Life Sciences, Scuola Superiore Sant'Anna, 56127, Pisa, Italy; 11 Institute of Clinical Physiology, National Research Council, 56124, Pisa, Italy; 12 Istituto Toscano Tumori, 56124, Pisa, Italy; 13 Laboratory of Post Genomic Molecular Genetic Research, Institute of Biochemical Physics, Russian Academy of Sciences, 119334, Moscow, Russia; 14 Department of Microbiology, Tumor and Cell Biology, Karolinska Institute, SE-17177, Stockholm, Sweden; Sapporo Medical University, JAPAN

## Abstract

The *SEMA3B* gene is located in the 3p21.3 LUCA region, which is frequently affected in different types of cancer. The objective of our study was to expand our knowledge of the *SEMA3B* gene as a tumor suppressor and the mechanisms of its inactivation. In this study, several experimental approaches were used: tumor growth analyses and apoptosis assays *in vitro* and in SCID mice, expression and methylation assays and other. With the use of the small cell lung cancer cell line U2020 we confirmed the function of *SEMA3B* as a tumor suppressor, and showed that the suppression can be realized through the induction of apoptosis and, possibly, associated with the inhibition of angiogenesis. In addition, for the first time, high methylation frequencies have been observed in both intronic (32-39%) and promoter (44-52%) CpG-islands in 38 non-small cell lung carcinomas, including 16 squamous cell carcinomas (SCC) and 22 adenocarcinomas (ADC), and in 83 clear cell renal cell carcinomas (ccRCC). Correlations between the methylation frequencies of the promoter and the intronic CpG-islands of *SEMA3B* with tumor stage and grade have been revealed for SCC, ADC and ccRCC. The association between the decrease of the *SEMA3B* mRNA level and hypermethylation of the promoter and the intronic CpG-islands has been estimated in renal primary tumors (*P* < 0.01). Using qPCR, we observed on the average 10- and 14-fold decrease of the *SEMA3B* mRNA level in SCC and ADC, respectively, and a 4-fold decrease in ccRCC. The frequency of this effect was high in both lung (92-95%) and renal (84%) tumor samples. Moreover, we showed a clear difference (*P* < 0.05) of the *SEMA3B* relative mRNA levels in ADC with and without lymph node metastases. We conclude that aberrant expression and methylation of *SEMA3B* could be suggested as markers of lung and renal cancer progression.

## Introduction

Semaphorins are negative mediators of axonal guidance in the central nervous system [1]. Semaphorins comprise a large family of glycoproteins (8 classes, including 5 vertebrate classes, of more than 30 members), but only class 3 (SEMA3) represents secreted soluble molecules. Members of the SEMA family are differentially expressed in cancer, and either promote or suppress cell proliferation, migration and angiogenesis, and the induction of drug resistance. Thus, the roles of separate members of semaphorin family can be quite different [2–9].

Class 3 semaphorins (SEMA3s, also known as collapsins) comprise one of five vertebrate families of semaphorins and play an important role in tumor biology, including regulating cellular processes, such as endothelial cell proliferation, apoptosis, migration and angiogenesis [10]. Recently, the involvement of this protein class in carcinogenesis has been intensively studied. SEMA3s are secreted by cells of multiple lineages, including epithelial cells, neurons, and specific tumor cells [10]. Neuropilins (NRP) represent the primary receptors of SEMA3s. The binding of SEMA3s to NRP1/2 initiates their downstream signaling but prevents the interaction between NRP1/2 and vascular endothelial growth factor (VEGF) and the subsequent induction of a pro-angiogenic transcriptional program. However, it is not clear whether SEMA3s inhibit tumor growth by competing with VEGF for neuropilins ligand-binding sites, by acting independently of VEGF, or by a combination of these effects [10–13].

Previous studies, including ours, of human chromosome 3 in renal, lung, breast and cervical carcinomas revealed frequent allelic losses (up to 40%) in the LUCA region (3p21.3), which harbors two semaphorins—SEMA3B and SEMA3F. This region (hg38/chr3: 50.0–50.5Mb) comprised of 445 Kb contains about 20 tumor suppressors (TSG) and TSG-candidates: *RASSF1*, *NPRL2*, *TUSC2*, *CACNA2D2* and others. Surprisingly, these genes playing roles in cellular processes and exerting tumor suppression by several different ways (cell cycle block, inhibition of angiogenesis, induction of apoptosis etc.) are located in the compact region [14–18].

Important evidence of tumor suppressor activity includes the identification of cell regulatory pathways and other mechanisms that are affected by SEMA3B. Using MDA-MB435 (breast carcinoma) and A549 (lung adenocarcinoma) cells it was previously shown that SEMA3B suppressed tumor growth but triggered a pro-metastatic program by releasing interleukin 8 [19, 20]. Furthermore, it was found that the induction of apoptosis by SEMA3B in tumor cells was mediated by inactivation of the Akt signaling pathway [21]. Therefore, it was important to further elucidate particular aspects of SEMA3B tumor suppression.

Methylation is an important mechanism of *SEMA3B* gene inactivation [17, 22]. However, the majority of previous research focused on methylation studies of the intronic CpG-island, that was incorrectly considered as located in the promoter region.

The objective of our study was to elucidate the distinct roles of SEMA3B in tumor suppression, particularly in apoptosis and angiogenesis. Furthermore we aimed to evaluate frequencies of promoter (hg38/chr3: 50,267,308–50,267,797) and intronic (hg38/chr3: 50,268,972–50,269,271) CpG-island hypermethylation correlations with *SEMA3B* expression, and tumor progression in lung and renal cancers.

## Materials and Methods

### Cell lines

Genomic DNA was isolated from 14 cancer cell lines: 3 squamous cell lung cancers (SCLC: ACC-LC5, NCI-N417, U2020), 2 non-small cell lung cancers (NSCLC: NCI-H157, NCI-H647) and 9 renal cell cancers (RCC: A498, ACHN, Caki-1, Caki-2, HN-51, KH-39, KRC/Y, TK-10, TK-164). The cell line U2020 was described earlier [23]. The ACC-LC5 cell line that carries a deletion in 3p21.3 [24] was kindly provided by Dr. Yusuke Nakamura (University of Tokyo, Tokyo, Japan). Renal A498, Caki1, and Caki2 and lung NCI-N417, NCI-H157, and NCI-H647 cell lines were purchased from the American Type Culture Collection (Manassas, VA, USA). Cell lines KRC/Y, ACHN, TK-164, HN-51, TK-10, and KH-39 were obtained from the Karolinska Institute (Stockholm, Sweden) cell line collection [25]. All human cell lines were grown as monolayer cultures in IMDM/RPMI or DMEM (with 4.5 g/l glucose) supplemented with 10% fetal calf serum (FCS).

### Tissue samples

In total, 70 paired tumor/normal samples of NSCLC (29 ADC and 41 SCC) and 133 clear cell RCC (ccRCC) were obtained from the N.N. Blokhin Cancer Research Center, Russian Academy of Medical Sciences (Moscow, Russia). The set of 38 NSCLC (16 ADC and 22 SCC) and 83 ccRCC was used in the methylation studies and the expression or copy number studies by semi-quantitative RT-PCR. The additional set of 32 NSCLC (13 ADC and 19 SCC) and 50 ccRCC was used for validation by qPCR expression studies. The sample information is presented in Table 1 and S1 Table. The samples were collected in accordance with the guidelines issued by the Ethics Committee of N.N. Blokhin Cancer Research Center, Russian Academy of Medical Sciences (Moscow, Russia). All patients gave written informed consent (available upon request). The Ethics Committee of N.N. Blokhin Cancer Research Center, Russian Academy of Medical Sciences, specifically approved this study. The study was performed in accordance with the principles outlined in the Declaration of Helsinki. Tumor tissues and paired morphologically normal tissues were obtained from patients after surgical resection prior to radiation or chemotherapy and were stored in liquid nitrogen. The diagnosis was verified by histopathology, and only samples with 70–80% or more tumor cells were used in the study. “Normal” controls were obtained at a minimum of 2 cm from the tumor and were confirmed histologically as normal epithelial cells. Tumor specimens were characterized according to the International System of Classification of Tumors, based on the tumor-node-metastasis (TNM) and staging classification of the Union for International Cancer Control (UICC, version 2002) [26] and World Health Organization (WHO) criteria classification [27, 28]. Blood samples from 15 healthy donors were also used in the study.

**Table 1:** Pathological and histological characteristics of the tumors.

Stage	Number of samples		
	ADC	SCC	ccRCC
**I**	6/7	7/6	20/22
**II**	2/1	8/6	23/12
**III**	7/5	7/7	30/14
**IV**	1/0	0/0	10/2
**All**	16/13	22/19	83/50

Note: The slash separates the number of samples used in the methylation studies and the expression or copy number studies by semi-quantitative RT-PCR and the number of samples used in the qPCR expression studies.

### SCID mice

Twelve SCID mice were used in the experiments. The mice were obtained from Scanbur (Sollentuna, Sweden). Animal euthanasia was performed by CO_2_ asphyxiation followed by cervical dislocation. This study was carried out in strict accordance with the recommendations in the Guide for the Care and Use of Laboratory Animals (NRC 2011), the European Convention for the Protection of Vertebrate Animals Used for Experimental and Other Scientific Purposes, Council of Europe (ETS 123), and the guidelines of the North Stockholm Ethical Committee for Care and Use of Laboratory Animals. The experiments with the SCID mice were approved by the North Stockholm Ethical Committee.

### Transfection and selection of stably transfected *SEMA3B*-U2020 cell clones

The cDNA encoding the *SEMA3B* gene was cloned into an episomal tetracycline—regulated vector, pETE. The resulting plasmid was sequenced. To obtain stable cell clones expressing *SEMA3B*, U2020 cells (SCLC) were transfected with empty pETE or pETE/*SEMA3B* plasmid DNA (0.5 mg DNA per well) in 12-well plates using Lipofectamine and Plus Reagent (Invitrogen, CA, USA) according to the manufacturer’s protocol. Transiently transfected pETE/*SEMA3B*-U2020 and pETE-U2020 cells were cultured for 2–3 weeks in IMDM medium containing Bsd (5 μg/ml) to select stable clones. The expression of *SEMA3B* was regulated by doxycycline.

### Colony formation assay

Transiently transfected U2020 cells (with pETE and pETE/*SEMA3B*) were stripped 24–48 h after transfection and plated on 100 mm^2^ cell culture dishes at a density of 500–1000 cells per plate. After selection with Bsd (5 μg/ml), Giemsa-stained colonies were photographed and counted using Quantity One software (version 4.4.0; Bio-Rad, Hercules, CA, USA). Cell viabilities were estimated by FACS analysis with propidium iodide (PI-FACS), following the manufacturer’s guidelines (FACSCalibur, BD Bioscience).

### Tumor growth in SCID mice

The tumorigenicity of pETE/*SEMA3B*-transfected U2020 and empty pETE-transfected U2020 cells (control) was assessed by subcutaneous injecting the cells into 6–8-week-old SCID mice as previously described [29]. The cells were collected by centrifugation at 800 rpm for 2 min and resuspended in serum-free IMDM medium at a concentration of 2–3×10^6^ cells per 100 μl injection. The cells were embedded into a Matrigel matrix (BD Biosciences, Erembodegem, Belgium) according to the manufacturer’s protocol. The mice were observed for tumor formation twice per week and the tumor size was measured using calipers. Then we used the multi-gene inactivation test (MGIT) for three genes (*ZMYND10/BLU*, *TUSC2/FUS1* and *SEMA3B*) in SCID mice. MGIT is based on the monitoring of tumor suppressor candidate gene inactivation in cells and tumors. It was accomplished according to a published method [29, 30]. Briefly, U2020 cells were transfected either with pETE/*SEMA3B*, pETE/*ZMYND10*, pETE/*TUSC2* or empty pETE plasmids. Mixes of cell clones were subcutaneously injected in SCID mice. Subsequently, if a tumor is formed, the expression of *SEMA3B*, *ZMYND10* and *TUSC2* is evaluated. The knock-down of the genes suggests their importance for tumor suppression.

### Immunohistochemical analysis of SCLC tumor cell line U2020 and tumor angiogenesis in SCID mice

The plasmid pETE/*SEMA3B* was introduced into SCLC cell line U2020, which constitutively produced a tetracycline transactivator (tTA) [29]. The resulting sub-line was inoculated subcutaneously into SCID mice. The mice were given drinking water with doxycycline (+dox, gene is OFF) or without (−dox, gene is ON). The mice were euthanized after 1 month. The tumors or places of cell injections were excised and embedded with paraffin. The sections were 5 μm in thickness. Paraffin was dissolved in xylene (Sigma-Aldrich, St. Louis, MO, USA), and the tissue sections were treated sequentially with 99, 95, 75 and 30% ethanol. Epitopes were recovered by heating in a microwave oven for 5 min in citrate buffer. Anti-CD31 mouse antibody, together with rabbit-anti-mouse FITC-conjugated secondary antibody (Dako, Karlstrup, Denmark), was used to stain microvessels. The TUNEL assay (In Situ Cell Death Detection Kit, Boehringer Mannheim, Germany) for the detection of apoptosis was performed according to the manufacturer’s protocol.

### DNA and total RNA isolation, reverse transcription-PCR

Nitrogen-frozen tissues were disrupted using a Mikro-Dismembrator (Sartorius, Germany). The DNA from human tissues and cell cultures was isolated by phenol extraction according to the standard protocols. Total RNA was isolated using the RNeasy mini kit as recommended by Qiagen (Netherlands). Purified RNA was quantified using NanoDrop-1000 (NanoDrop Technologies Inc., DE, USA). RNA quality was assessed using 28S and 18S rRNA bands after electrophoresis in a 1% denaturing agarose gel and analyzed using a Bioanalyzer 2100 (Agilent Technologies, CA, USA). The lack of DNA contamination was checked by semi-quantitative PCR with primers for the main histocompatibility complex I gene (*MHCI*, designed using Vector NTI, see S2 Table). All RNA samples were treated with RNase-free DNase I (Fermentas, Lithuania). RNA samples containing over 0.1% DNA were discarded. The cDNA was synthesized from 1 μg of total RNA using M-MuLV reverse transcriptase and random hexamers, according to the standard manufacturer’s protocol (Fermentas, Lithuania).

### Bisulfite sequencing of the promoter CpG-island of *SEMA3B* gene

Bisulfite DNA conversion was conducted as described in [31, 32] with the use of 1–2 μg DNA (lung and renal cell lines and tumor/normal tissues). The modified DNA was purified using a JETquick PCR Purification Spin Kit (Genomed, Sweden). Modified DNA was maintained at -20°C and used as a template for PCR with the designed primers (listed in S2 Table), whose product was sequenced. Amplification of the *SEMA3B* promoter CpG-island fragment was performed in a 50 μl reaction mixture containing PCR buffer (67 mM Tris-HCl pH 8.8, 16.6 mM ammonium sulfate, 0.01% Tween 20); 2.0 mM MgCl_2_; 0.25 mM of each dNTP; 25 pM of each primer; 1 unit Hot Start Taq DNA polymerase (SibEnzyme, Russia); and 5–20 ng of modified DNA in a DNA Engine Dyad Cycler (Bio-Rad, United States) using the following program: 95°C, 5 min; 35 cycles of 95°C, 15 s; 62°C, 30 s; 72°C, 30 s and 72°C, 7 min. The PCR amplified product was purified using 1.5% agarose gel electrophoresis and the JETquick Gel Extraction Spin Kit (Genomed, Sweden). For sequencing, 5–10 ng of the purified DNA fragment and 25 pM of one of the primers were used. Sequencing was conducted using an automatic sequencing machine (Beckman-Coulter).

### Methylation specific PCR (MSP)

The bisulfite-treated DNA, dissolved in twice-distilled water, was also used as a template for MSP. The PCR conditions and primers for the methylated and unmethylated allele of intronic [17] and promoter (designed by DNASTAR Lasergene 2000 program) CpG-islands are given in S2 Table. In case of the promoter CpG-island, 6 CpG-dinucleotides were analyzed (2 by the forward primer and 4 by the reverse), and in case of the intronic—3 (1 by the forward primer and 2 by the reverse). PCR was performed on a DNA Engine Dyad Cycler amplifier (Bio-Rad, United States) using the following program: 95°C, 5 min; 35 cycles of 95°C, 10 s; T_ann_ (see S2 Table), 20 s; 72°C, 30 s and 72°C, 3 min. The absence of PCR product on unconverted DNA was checked for each pair of primers. DNA of the human fibroblast cell line L-68 served as an unmethylated allele control; L-68 SssI DNA from L-68 fibroblasts treated with SssI methyltransferase (SibEnzyme, Russia) served as a positive control for 100% methylation.

### Semi-quantitative RT-PCR

To control the reverse transcription, primers for the transcript of the beta2-microglobulin (*B2M*) gene were used [33]. Semi-quantitative RT-PCR was performed with equal quantities of cDNA using the primers and conditions listed in S2 Table. Multiplex PCR with primers to the *SEMA3B* [17] and *B2M* genes was performed under conditions optimized for the *SEMA3B* primers, and the concentration of *B2M* primers was 1.5 times lower. The products of the RT-PCR were separated on 2% agarose gels, and the band pattern was analyzed using GelImager software (DNA Technology, Russia). A semi-quantitative copy number assay for the markers of LUCA region was used as described elsewhere [14].

### qPCR

Quantitative PCR was performed with the primers and TaqMan probes listed in S2 Table using a 7500 Real-Time PCR System (Applied Biosystems, CA, USA). Each reaction was repeated three times. The nucleotide sequences of the amplicons were verified by sequencing in a 3730 DNA Analyzer automated sequencer (Applied Biosystems, CA, USA). QPCR data were analyzed using the reference genes *GAPDH*, *GUSB* and *RPN1* [34] and the relative quantification or ΔΔCt-method as described earlier [35]. At least 2-fold mRNA level changes were considered as significant because of mRNA level variability of the reference genes.

### Statistical analysis

The nonparametric Wilcoxon test was used to compare mRNA expression differences in the target and reference genes in the ccRCC and NSCLC samples. Kruskal-Wallis and Mann-Whitney rank-sum tests, Fisher’s exact test and χ^2^ criteria were used for analysis of mRNA level and methylation status changes in ccRCC and NSCLC groups with different pathological and histological characteristics. Student's t-test was used to compare the data obtained for groups of replicates. P-values ≤ 0.05 were considered statistically significant. Spearman’s rank correlation coefficient (*r*_*s*_) was used for revealing correlations.

## Results

### *In vitro* growth suppression of SCLC cells U2020 by *SEMA3B*

**Fig 1:**
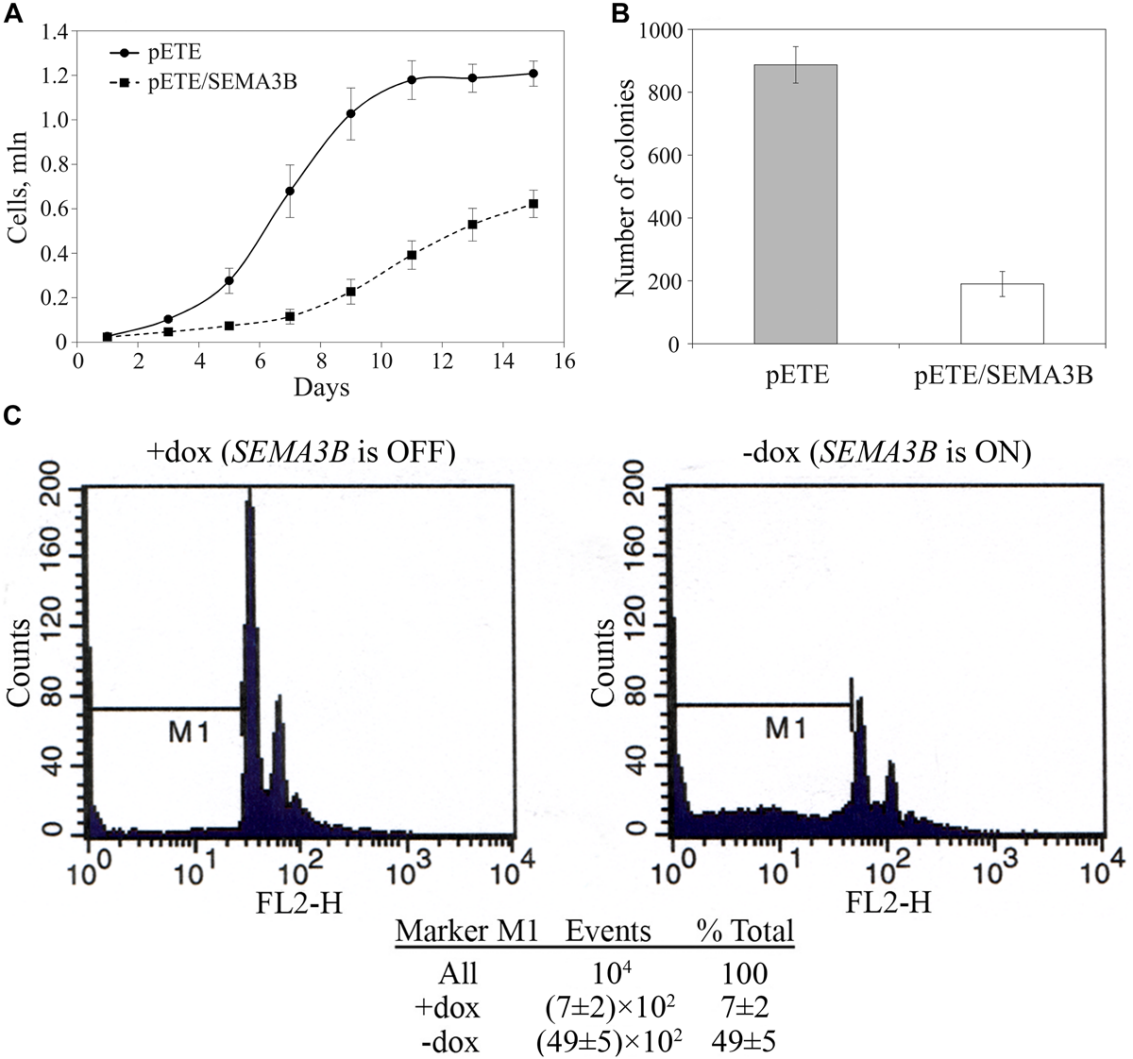
*In vitro* growth of U2020 cells (U7111 clone) depends on the expression of *SEMA3B*. A—The growth rate of U2020 cells: dashed line with squares—U7111 clone with pETE/*SEMA3B*, solid line with circles—U2020 cells with pETE (control); B—colony formation assay; C—PI-FACS analysis of cells with and without expression of *SEMA3B*. Mean values ± standard deviations for 4 replicates are represented in each case.

The small-cell lung carcinoma cell line U2020 was transfected with pETE*/SEMA3B* or pETE as the control. The transfected cells were cultured for 15 days. The growth rate of U2020 cells expressing *SEMA3B* was lower than the control (*P* < 0.01 since the day 5, see Fig 1A). The colony formation assay showed that the number of colonies of U2020 cells containing pETE/*SEMA3B* was lower after re-expression of the doxycycline-suppressed *SEMA3B* gene in comparison to the control cells (890 ± 60 vs 190 ± 40, *P* < 0.01, Fig 1B). Based on PI-FACS analysis, the abundance of apoptotic and necrotic cells expressing *SEMA3B* (without doxycycline) was increased significantly in comparison with *SEMA3B*-off cells (with doxycycline): from (7±2)×10^2^ to (49±5)×10^2^, *P* < 0.01, see Fig 1C. Taken together, these data suggest that *SEMA3B* is the inhibitor of human SCLC cells growth via induction of apoptosis *in vitro*.

### Multi-gene inactivation test in SCID mice

We have previously reported that the GIT technique allows for the efficient and controlled induction of various genes in cells [29, 30]. For the MGIT experiment, we used the SCLC cell line U2020 for the conditional expression of three TSG-candidates located in 3p21.3: *ZMYND10* (*BLU*), *TUSC2* (*FUS1*) and *SEMA3B*. Mixes of cell clones carrying different genes were inoculated subcutaneously into six-week-old SCID mice using a Matrigel (basement membrane matrix) implantation technique in the absence of doxycycline (the genes were ON).

**Fig 2:**
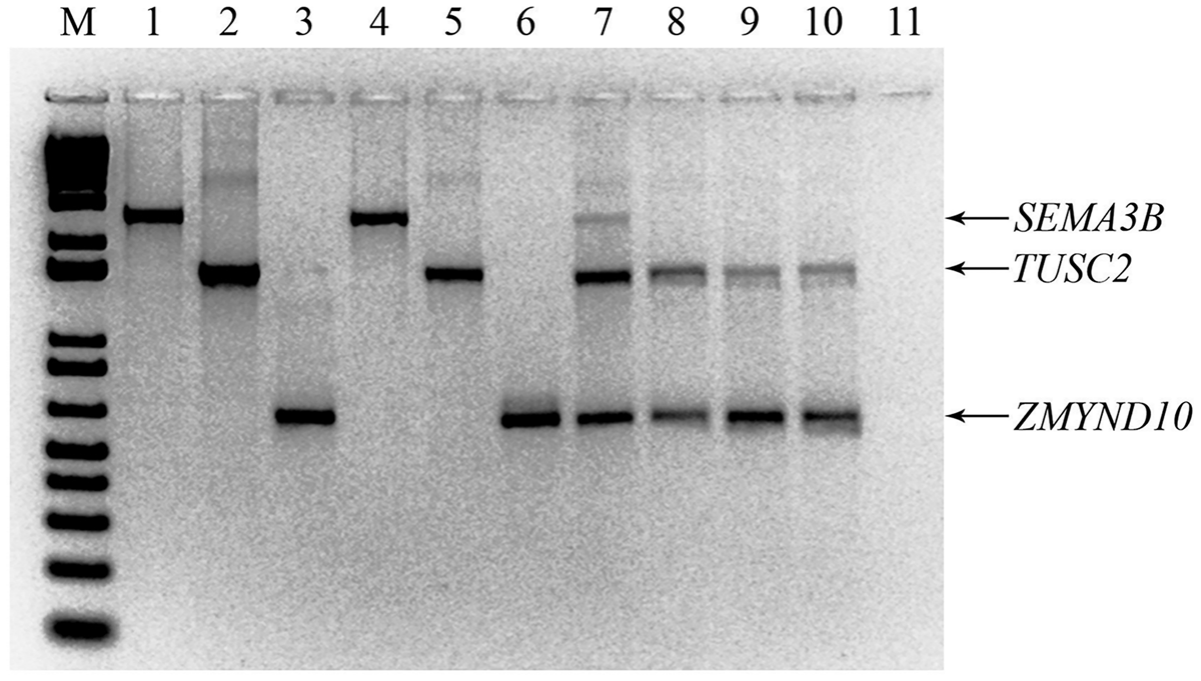
Absence of *SEMA3B* expression in tumors grown *in vivo*. Electropherogram of multiplex PCR from plasmids, clones and SCID mice tumors of three genes. M—marker, 1—PCR from plasmid pETE/*SEMA3B*, 2—PCR from plasmid pETE/*TUSC2*, 3—PCR from plasmid pETE/*ZMYND10*, 4—PCR from U7111/*SEMA3B* cell clone 1, 5—PCR from U7111/*TUSC2* cell clone 3, 6—PCR from U7111/*ZMYND10* cell clone 4, 7—mixed cell clones, 8—PCR from tumor 1, 9—PCR from tumor 2, 10—PCR from tumor 3, 11—negative control.

A PCR-based comparison of SCID mice tumors and primary cell clones showed unequivocally that *SEMA3B* was selectively knocked-down in all three tumors grown *in vivo* (see samples 8–10 in Fig 2), whereas the expression of *ZMYND10* and *TUSC2* genes did not change under these conditions. These two genes most likely did not antagonize the tumor growth of U2020 cells in SCID mice (we leave this question open because the search for mutation through retained genes was not included in the MGIT). These data suggest that SEMA3B is a growth inhibitor of human SCLC cells *in vivo*.

### Effect of *SEMA3B* transgenes on tumor growth in SCID mice and angiogenesis

**Fig 3:**
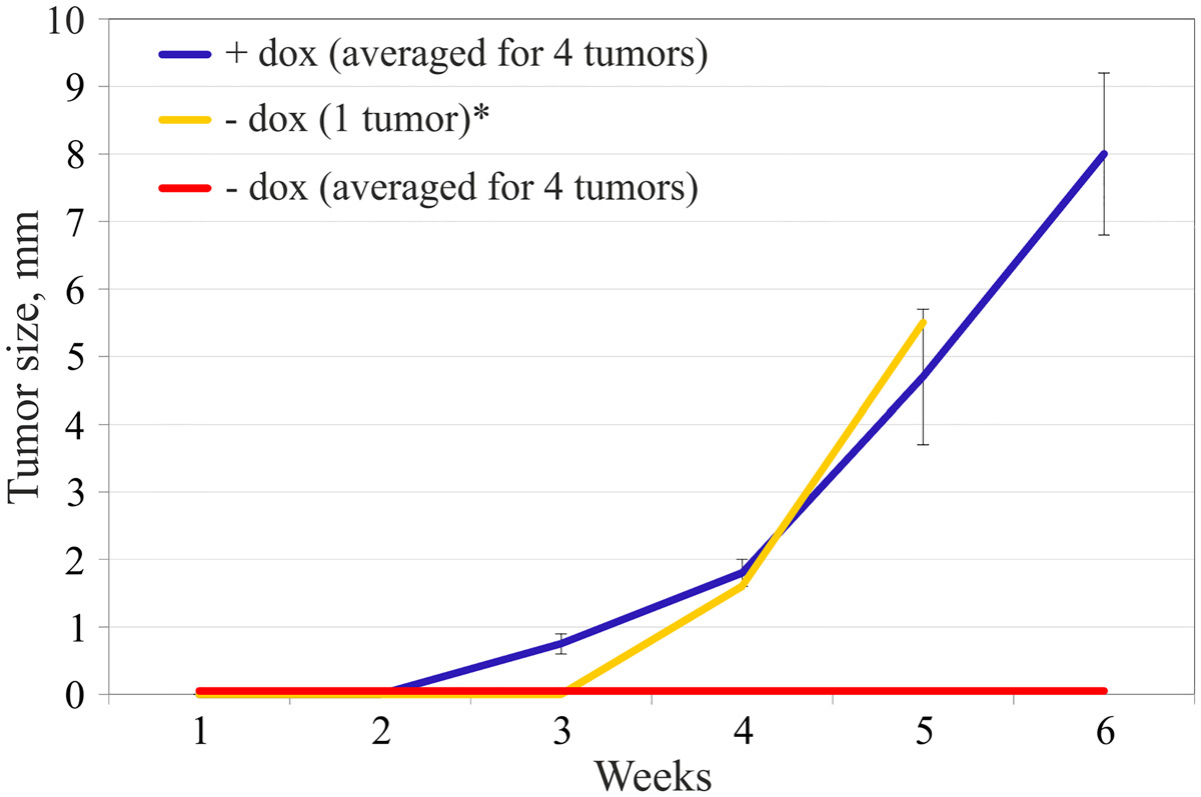
Inhibition of tumor growth by *SEMA3B* re-expression. The growth rate of U2020 cells (U7111 clone) in SCID mice: blue line—U2020 cells without *SEMA3B* expression (+ doxycycline, 4 mice), red and yellow line—U2020 cells with *SEMA3B* expression (- doxycycline, 4 mice and 1 mouse respectively). *—no expression of *SEMA3B* gene according to the Northern blot (data not shown). One +dox and one—dox mice were withdrawn from the study after one month.

The U2020 sub-line with conditional *SEMA3B* expression under doxycycline control was inoculated in SCID mice subcutaneously. Four mice received doxycycline in drinking water (+dox mice, control) and five were not administered doxycycline (-dox mice). The onset of solid tumors actively expressing *SEMA3B* was not observed in 4 out of 5 cases (Fig 3, red line). In the fifth—dox mouse active tumor growth (Fig 3, yellow line) began one week later compared to the control (Fig 3, blue line), in spite of the presence of *SEMA3B* in the construct. However, the expression of the *SEMA3B* gene was not detected in this tumor according to the Northern blot analysis (data not shown) suggesting loss of *SEMA3B* in these cells. The tumors (observed in *SEMA3B*-OFF mice) had areas of active cell proliferation, in contrast to the tissues taken from sites of cell injections (in *SEMA3B*-ON mice), in which abundant fibrous and poorly differentiated cellular stroma and necrotic areas were observed. These data demonstrate the possibility of *SEMA3B* tumor suppression activity in SCID mice.

**Fig 4:**
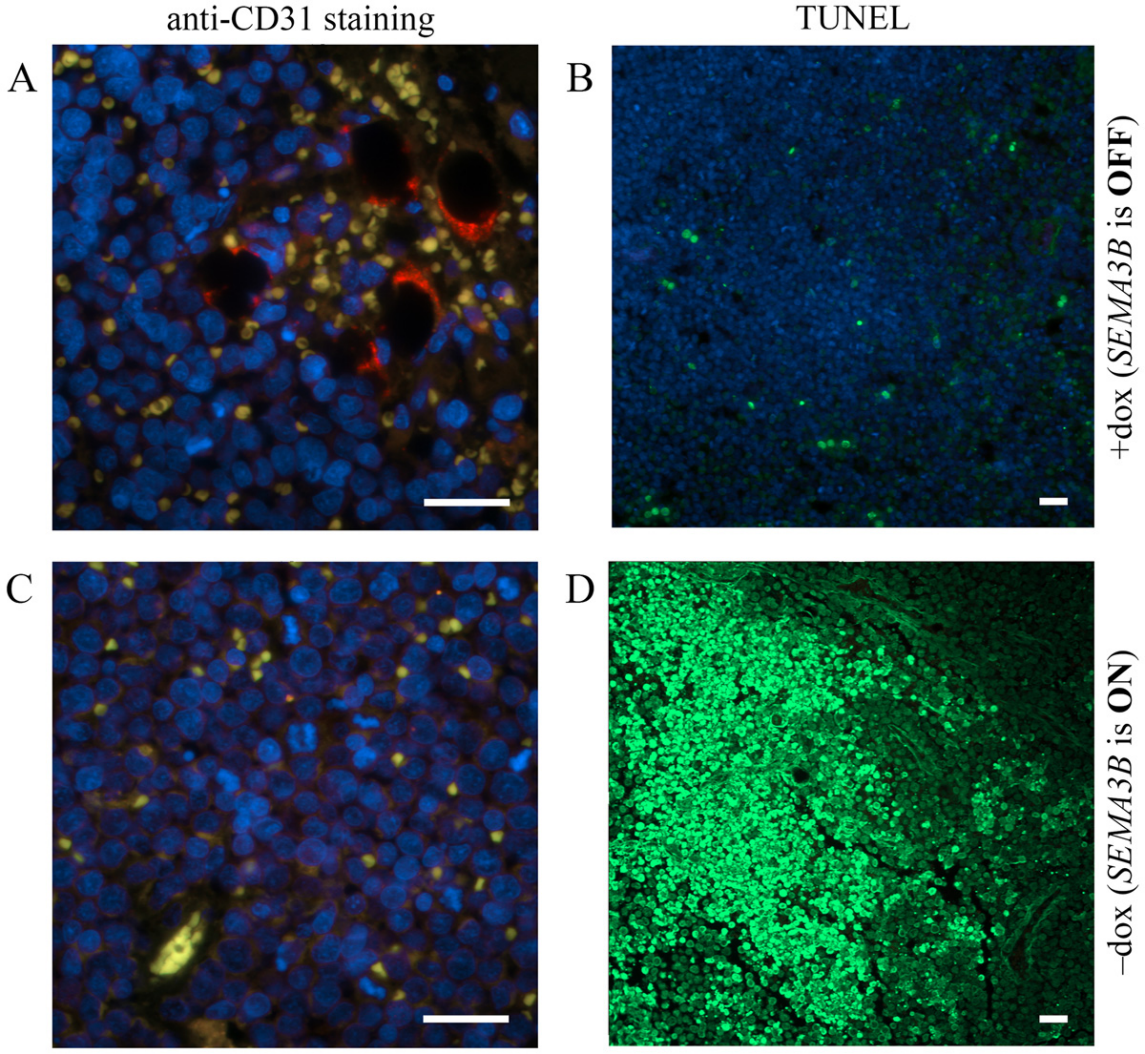
Tumor sections from SCID mice. (A, B)—*SEMA3B* is OFF (mouse received doxycycline in drinking water). (C, D)—*SEMA3B* is ON (mouse were not administered doxycycline). (A, C)—staining with anti-CD31 to monitor blood vessels (green signal). When *SEMA3B* is OFF, areas filled with erythrocytes (red signal) are seen. Yellow signal indicates co-localization of green and red signals. Blue signal corresponds to DNA. (B, D)—TUNEL assay. Notice the area with apoptotic cells (green signal) where the *SEMA3B* gene was expressed.

Tissues from two sites of U2020 cells inoculation were analyzed using CD31 staining and TUNEL assay: one SEMA3B-negative (Fig 4A and 4B) and one SEMA3B-positive (Fig 4C and 4D) mouse. Anti-CD31 mouse antibodies were used to stain blood microvessels. A very few number of microvessels was detected in SEMA3B-positive tissues. A fragment containing one of the microvessel-like objects is shown at Fig 4C. Microvessel signal (green channel) was co-localized with signal from erythrocytes (red channel) resulting to yellow colored areas in Fig 4A. However, SEMA3B-negative solid tumors demonstrated abundance of elongated, compressed blood ducts with fluorescence typical to epithelia. These tumor ducts were also co-localized with erythrocytes (Fig 4A). This could support a role of SEMA3B as inhibitor of angiogenesis. However, further experiments on the extended sampling are needed to prove this suggestion.

We hypothesized that the SEMA3B-positive cells in the areas without blood vessels and erythrocytes were undergoing cell death. To test this hypothesis, sections of the same tissue fragments were analyzed by a TUNEL assay, a technique enabling detection of apoptotic cells. (Fig 4B, 4D). A huge area of apoptotic cells was observed in SEMA3B-positive tissue sample whereas no apoptotic area was seen in SEMA3B-negative tumors. In this case, we assumed that the expression of *SEMA3B* suppressed tumor growth *in vivo*, likely by the induction of apoptosis. Inhibition of angiogenesis could be suggested also.

### Methylation of the promoter and intronic *SEMA3B* CpG-islands in lung and renal cell lines and primary tumors by bisulfite sequencing and methylation-specific PCR

**Fig 5:**
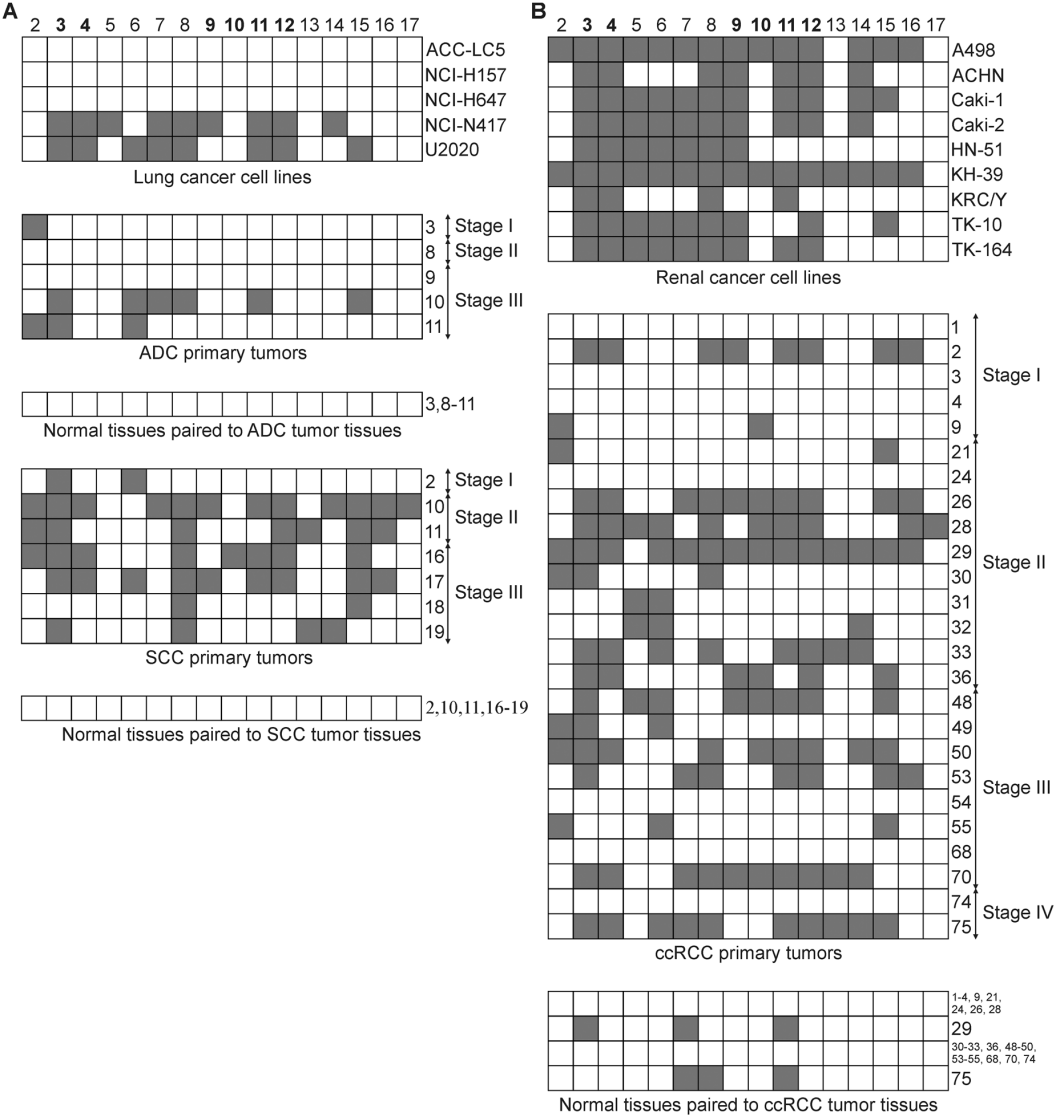
Methylation profile of the promoter CpG-island of the *SEMA3B* gene in lung (A) and renal (B) cancer cell lines and primary tumors. Bisulfite sequencing data, 16 CpG-dinucleotides (2–17) of the CpG-island are given. Grey squares show methylated CpG-dinucleotides, white squares—unmethylated. Numbers of primary tumors correspond to those in S1 Table. The bold numbers of CpG-dinucleotides (3–4 and 9–12) indicate the location of the primers that were used for MSP method.

The *SEMA3B* gene is comprised of 18 exons and contains two CpG-islands: one is located in the promoter region (1-st CpG-island, hg38/chr3: 50,267,308–50,267,797, 22 CpG-dinucleotides) and the other one in the first intron (2-nd CpG-island, hg38/chr3: 50,268,972–50,269,271, 12 CpG-dinucleotides). We analyzed the methylation profile of 16 CpG-dinucleotides of the promoter CpG-island in 5 lung cancer cell lines (3 SCLC and 2 NSCLC) and 12 NSCLC primary tumors using bisulfite sequencing (5 ADC and 7 SCC, see Fig 5A). Dense methylation (> 40% of the analyzed CpGs were methylated) of the promoter CpG-island of the *SEMA3B* gene was observed in 2 of 3 SCLC cell lines, but in none of the NSCLC cell lines (NCI-H157 and NCI-H647). However, in all 7 SCC primary tumors and in 2 of 5 ADC primary tumors, methylation was detected in 2–12 of the CpGs. In addition, we examined the methylation profile in 9 renal cancer cell lines and 25 ccRCC primary tumors (Fig 5B). Dense methylation of the promoter CpG-island was observed in 8 of 9 cell lines and 11 of 25 ccRCC primary tumors. Among matched histological normal tissues, methylated CpGs were detected only in 2 of 25 ccRCC cases and in none of 12 NSCLC cases (Fig 5A and 5B).

Next, we evaluated the methylation frequencies of both CpG-islands of the *SEMA3B* gene by the MSP method using a representative set of primary tumors. The methylation frequency of the promoter CpG-island was 44% (7/16) in ADC, 45% (10/22) in SCC and 52% (43/83) in ccRCC. The intronic CpG-island was methylated slightly less than the promoter island in both histological types of NSCLC (ADC −38%, 6/16; SCC −32%, 7/22) and in ccRCC (39%, 32/83; Table 2). The methylation frequencies of both islands were significantly higher in tumor tissues than in paired histologically normal tissues (*P* ≤ 0.04, see Table 2). Thus, the methylation of both CpG-islands of the *SEMA3B* gene is a hallmark of lung and renal cancer. As expected, the methylation of neither the 1-st nor the 2-nd *SEMA3B* CpG-island was detected in any DNA samples isolated from blood lymphocytes of healthy donors (n = 15). The MSP data were in agreement with the bisulfite sequencing results for each type of cancer investigated.

**Table 2:** Methylation frequencies of two CpG-islands of the *SEMA3B* gene in NSCLC (ADC and SCC) and ccRCC in groups of samples with different pathological and histological characteristics.

Stage	ADC				SCC				ccRCC			
	1-st CpG		2-nd CpG		1-st CpG		2-nd CpG		1-st CpG		2-nd CpG	
	T	N	T	N	T	N	T	N	T	N	T	N
**I**	33%	0%	33%	0%	14%	14%	0%	0%	40%	0%	45%	0%
	(2/6)	(0/6)	(2/6)	(0/6)	(1/7)	(1/7)	(0/7)	(0/7)	(8/20)	(0/20)	(9/20)	(0/20)
**II**	50%	0%	50%	0%	75%	13%	38%	0%	44%	4%	30%	9%
	(1/2)	(0/2)	(1/2)	(0/2)	(6/8)	(1/8)	(3/8)	(0/8)	(10/23)	(1/23)	(7/23)	(2/23)
**III**	43%	14%	29%	0%	43%	0%	57%	0%	60%	3%	33%	17%
	(3/7)	(1/7)	(2/7)	(0/7)	(3/7)	(0/7)	(4/7)	(0/7)	(18/30)	(1/30)	(10/30)	(5/30)
**IV**	100%	0%	100%	0%	-	-	-	-	70%	10%	60%	0%
	(1/1)	(0/1)	(1/1)	(0/1)					(7/10)	(1/10)	(6/10)	(0/10)
**All**	44%	6%	38%	0%	45%	9%	32%	0%	52%	4%	39%	8%
	(7/16)	(1/16)	(6/16)	(0/16)	(10/22)	(2/22)	(7/22)	(0/22)	(43/83)	(3/83)	(32/83)	(7/83)
	*P = 0*.*04*		*P = 0*.*02*		*P = 0*.*02*		*P = 0*.*01*		*P = 6×10* ^*–13*^		*P = 6×10* ^*–6*^	

Note: MSP data. 1-st CpG—promoter CpG-island; 2-nd CpG—intronic CpG-island; T—primary tumors; N—morphologically normal (conventional “normal”) tissues paired to tumor tissues. The P-values show the significance of the methylation frequencies distinction between tumor and normal tissues (Fisher’s exact test and χ^2^ criteria).

The use of a representative set of primary tumors allowed us to reveal possible correlations between the methylation frequency of the promoter and intronic CpG-islands with the pathological and histological parameters of the tumors. Advanced ccRCC and NSCLC tumors had a higher frequency of CpG-island methylation compared to the early stages (Tables 2 and 3). The strongest correlation was shown for SCC (Spearman’s rank correlation coefficients were equal 0.37, *P* = 0.09, and 0.60, *P* < 0.01, for the 1-st and for the 2-nd CpG-island, respectively). A positive correlation was observed between tumor grade and the frequency of CpG-island methylation for NSCLC and ccRCC (Table 3). This correlation was stronger than the correlation between tumor stage and the frequency of CpG-island methylation for both histological types of NSCLC and almost equal to those for ccRCC (Table 3). The strongest correlation was shown for SCC (0.49, *P* = 0.02, and 0.68, *P* < 0.01, for the 1-st and for the 2-nd CpG-island, respectively). These results suggest that methylation of both *SEMA3B* CpG-islands is a frequent event in renal and lung cell lines and primary tumors and contributes to the progression of tumors of these locations, especially in the lung.

**Table 3:** Correlations between the frequency of CpG-island methylation and tumor stage or grade.

Feature		ADC		SCC		ccRCC	
		1-st CpG	2-nd CpG	1-st CpG	2-nd CpG	1-st CpG	2-nd CpG
**Stage**	*r*_*s*_	0.36	0.30	0.37	0.60	0.34	0.21
	*P*	*0*.*17*	*0*.*26*	*0*.*09*	*< 0*.*01*	*< 0*.*01*	*0*.*05*
**Grade**	*r*_*s*_	0.46	0.37	0.49	0.68	0.33	0.33
	*P*	*0*.*07*	*0*.*16*	*0*.*02*	*< 0*.*01*	*< 0*.*01*	*< 0*.*01*

Note: Based on MSP data. 1-st CpG—promoter CpG-island; 2-nd CpG—intronic CpG-island; *r*_*s*_—Spearman’s rank correlation coefficient; *P*—p-value.

### Correlation between methylation status of two CpG-islands and *SEMA3B* gene expression level in ccRCC tumors

**Fig 6:**
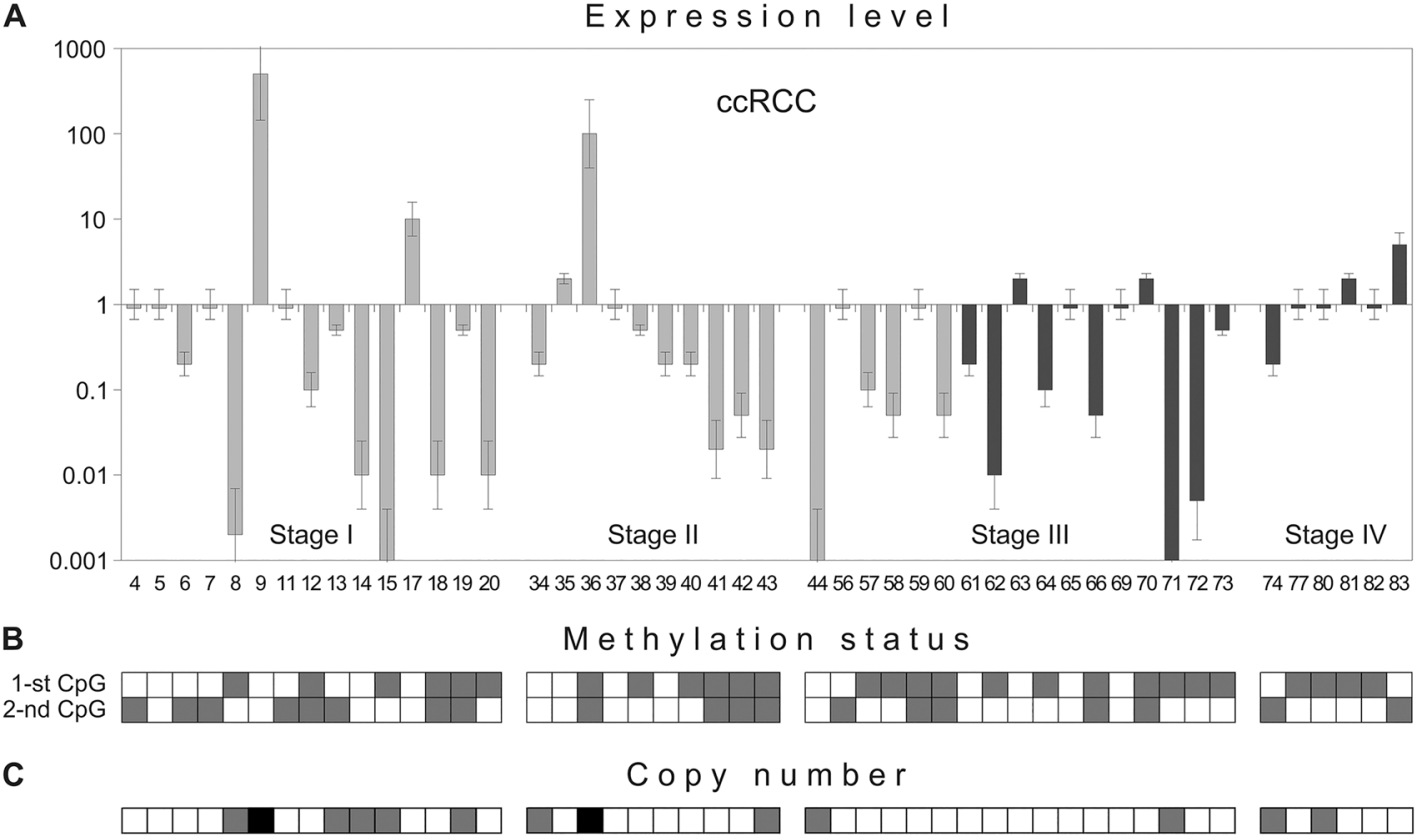
*SEMA3B* gene expression level (A), copy number (C) and methylation status of its two CpG-islands (B) in the same ccRCC samples. Semi-quantitative PCR (A, C) and MSP (B) data. Numbers of primary tumors correspond to those in S1 Table and Fig 5B. (A) Light grey columns—samples without metastases, dark grey columns—samples with lymph node or distant metastases. (B) 1-st CpG—promoter CpG-island, 2-nd CpG—intronic CpG-island. Grey squares show methylated CpG-islands, white squares—unmethylated. (C) Grey squares show hemi- or homozygous deletions of the 5’Sema5 marker, black—amplification, white squares—retention. Assessed mean values ± error bars are represented in the “A” part.

To investigate the consequences of CpG-island methylation, the mRNA level in 48 ccRCC samples was evaluated by semi-quantitative PCR. In ccRCC samples, the expression of *SEMA3B* was 5–1000 times lower than the matched normal samples in 24 of 48 cases (Fig 6A). In 4 cases, a 5-300-fold up-regulation of expression was observed. No correlation between mRNA level changes and stage, grade or presence of metastases was observed.

Next, we compared the changes in mRNA levels of the *SEMA3B* gene in ccRCC tumors (semi-quantitative PCR data) with the methylation status of two CpG-islands (methylation-specific PCR data). Methylation of the promoter CpG-island was observed in 27 of 48 (56%) cases and methylation of the intronic CpG-island—in 19 of 48 (40%) (Fig 6B). As shown in Fig 6A and 6B, both islands were methylated primarily in the ccRCC samples with decreased *SEMA3B* expression. The Spearman’s correlation coefficient between expression level down-regulations and methylation status was equal to 0.50 (*P* < 0.01) for the 1-st CpG-island and 0.25 (*P* < 0.01) for the 2-nd CpG-island. These data suggest that inactivation of *SEMA3B* in carcinomas could be associated primarily with methylation of the promoter CpG-island.

### Amplifications and deletions of the *SEMA3B* gene can contribute to mRNA level alterations in ccRCC

A correlation between changes in *SEMA3B* mRNA expression and the methylation status of its promoter CpG-island was shown in ccRCC, but for several samples, significant aberrations in expression were not associated with methylation of either the 1-st or the 2-nd CpG-island. In one sample, a 100-fold up-regulation was observed when both CpG-islands were methylated. Consequently, we investigated an alternative mechanism of expression regulation, copy number alterations, by semi-quantitative PCR. The increase in *SEMA3B* mRNA level in ccRCC samples #9 and #36 (300- and 100-fold up-regulation, respectively) was associated with amplification of the 5’Sema5 marker [14] (Fig 6A and 6C). A five-fold increase in mRNA level in ccRCC sample #83 was associated with amplification of the D3S1573 marker (data not shown). Decreased *SEMA3B* expression (100–1000 times) in ccRCC samples #8, #14, #15, #44 and #71 was associated with hemi- or homozygous deletions of the 5’Sema5 marker (Fig 6A and 6C). A hemizygous deletion was observed in sample #34 with 5-fold down-regulation and no methylation. The Spearman’s correlation coefficient between expression level alterations and copy number changes was equal to 0.61 (*P* < 0.01) Thus, in addition to the epigenetic modifications, genetic events (e.g., deletion or amplification of the *SEMA3B* gene locus) can also contribute to the alteration of *SEMA3B* gene expression in tumors. However, in samples #39 and #61, with 4-5-fold down-regulation, and sample #17, with a 10-fold up-regulation, no copy number changes were observed. We propose that other mechanisms may be responsible for these mRNA level alterations; for example, regulation via miRNA.

### Quantitative evaluation of *SEMA3B* expression in lung and renal tumors

**Fig 7:**
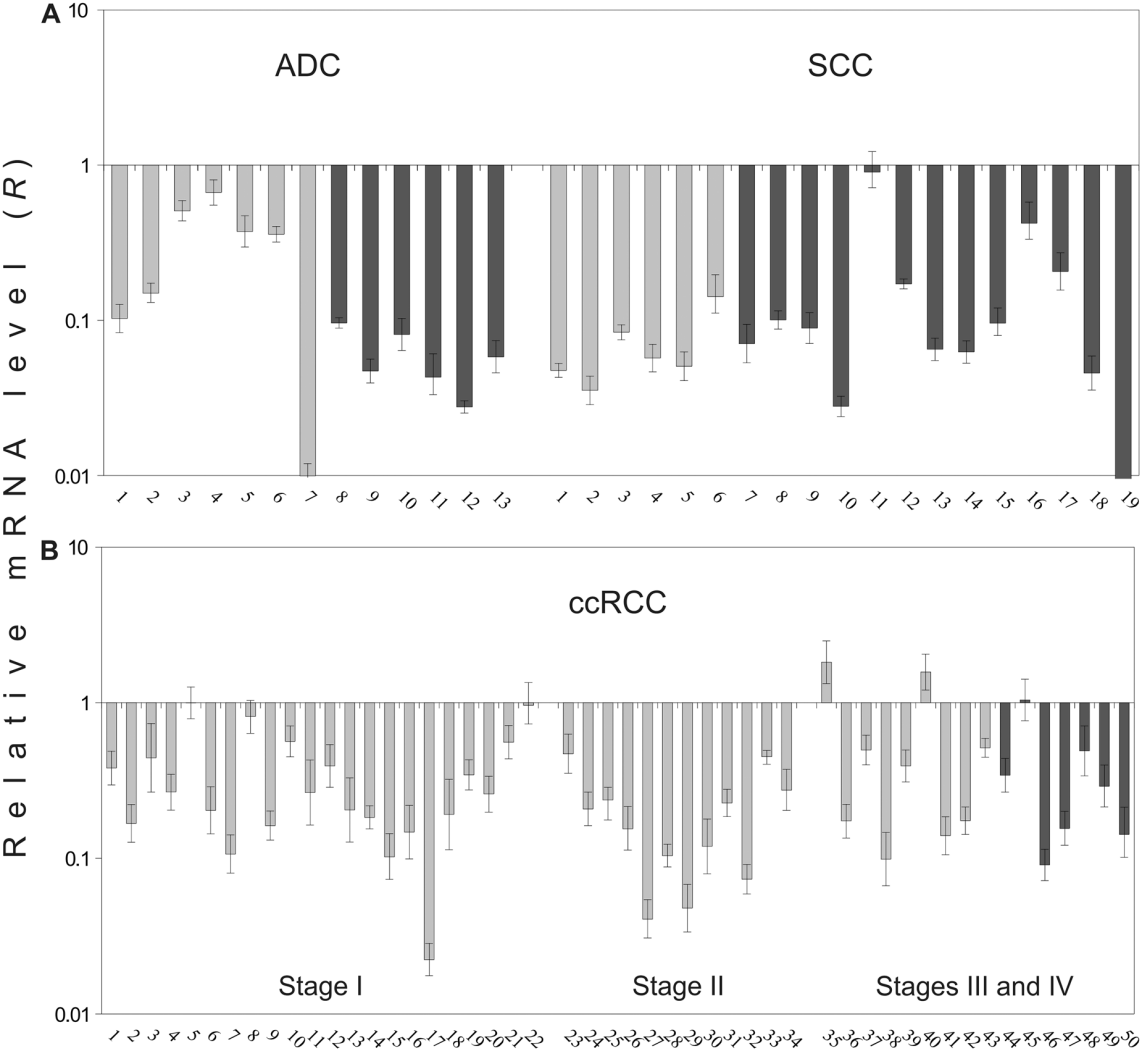
Relative mRNA level of the *SEMA3B* gene in NSCLC (A) and ccRCC (B). QPCR data, additional samplings. Light grey columns—samples without metastases, dark grey columns—samples with lymph node or distant metastases. The numbers of primary tumors correspond to those in S1 Table. Mean values ± standard deviations for 3 replicates are represented.

To confirm the mRNA level differences, we performed qPCR analysis on an additional set of lung and renal tumors (Table 1). We found a noticeable (up to 300 times) and frequent (94%, 30/32) down-regulation of *SEMA3B* gene expression in NSCLC primary tumors using qPCR (Fig 7A). The overall frequency and extent of the decrease in *SEMA3B* mRNA level was similar in the tumors of the two main histological types of NSCLC; ADC and SCC. An average 10-fold decrease and 92% (12/13) was observed in ADC, and a 14-fold and 95% (18/19) was observed in SCC (Table 4). Other differences were also observed. In ADC with lymph node metastases compared to ADC without metastases, the average mRNA level was considerably decreased (19-fold decrease vs. 3-fold, *P* < 0.05), but in SCC, significant expression down-regulation was observed in the early stages of tumor development.

**Table 4:** Frequency and mRNA level changes of the *SEMA3B* gene in NSCLC (ADC and SCC) and ccRCC in groups of samples with different pathological and histological characteristics.

Stage	Median of *R*, n-fold decrease*			Frequency of *R* decrease, %		
	ADC	SCC	ccRCC	ADC	SCC	ccRCC
**I**	3	19	4	86	100	77
	(1–10^2^)	(7–28)	(1–45)	(6/7)	(6/6)	(17/22)
**II**	10	11	6	100	83	100
		(1–36)	(2–25)	(1/1)	(5/6)	(12/12)
**III+IV**	21	15	3	100	100	81
	(12–36)	(2–3×10^2^)	(1–11)	(5/5)	(7/7)	(13/16)
**All**	10	14	4	92	95	84
	(1–10^2^)	(1–3×10^2^)	(1–45)	(12/13)	(18/19)	(42/50)

Note: QPCR data. *R*—relative mRNA level.

*—in parentheses a range of mRNA level changes is shown.

In ccRCC samples we also observed frequent (84%, 42/50) *SEMA3B* gene expression down-regulation (4-fold on average, Fig 7B). However, these values were not as high as for NSCLC. No correlation was observed between mRNA level changes and stage (Table 4) or the presence of metastases (Fig 7B).

Based on these results, we conclude that significant and frequent decreases in *SEMA3B* mRNA level are in agreement with the semi-quantitative PCR and methylation data and confirm that *SEMA3B* down-regulation is a frequent event both in NSCLC and ccRCC. However, the frequency of down-regulation was higher than the frequency of CpG-island methylation (Tables 2 and 4). Thus, other mechanisms of inactivation are also likely contributors: for example, deletions in renal, lung and breast cancers, according to our results and the data of others [14, 15, 17].

## Discussion

The *SEMA3B* gene is located in the LUCA region (3p21.3) that harbors 19 genes, the majority of which are involved in carcinogenesis [16, 36–38]. Some of these genes, e.g., *RASSF1*, *NPRL2*, and *SEMA3B*, suppress the growth of tumor cells *in vitro* and *in vivo* [36–38]. However, *SEMA3B* may have both tumor-suppressive and pro-invasive properties. It has been reported that *SEMA3B* is dramatically down-regulated in the cell line H460-M, derived from the large cell lung tumor cell line NIH-H460, which induces spontaneous metastasis in nude mice [39]. On the other hand, it is highly expressed in many invasive and metastatic human cancers; for example, colorectal carcinoma, neuroblastoma, melanoma and acute myeloid leukemia [20, 40]. SEMA3B induces apoptosis and the production of interleukin 8 by tumor cells by initiating the p38-mitogen-activated protein kinase pathway. In turn, the release of interleukin 8 induces the recruitment of tumor-associated macrophages, which may lead to metastatic dissemination of the tumor [20, 41]. In this study, we confirmed the tumor-suppressive activity of SEMA3B both *in vitro* (U2020 SCLC cells) and *in vivo* (SCID mice models, by both tumor growth assay and multi-gene inactivation tests). Our studies *in vitro* are in agreement with previous research in NSCLC, breast and ovarian ADC cell lines [19, 22, 42]. However, in this study, the suppressive effect of SEMA3B *in vivo* has been shown for the first time.

Semaphorins 3 encode proteins with axonal guidance properties that generally affect the motility and migration of tumor and endothelial cells by inducing a collapse of the actin cytoskeleton via binding to neuropilins/plexins complexes [43, 44]. Earlier, the class-3 semaphorins were implicated in tumor progression and metastasis [39, 45, 46]. However, tumor suppression properties of distinct semaphorins differ across various cancer types. For example SEMA3D and SEMA3E displayed strong inhibitory effect on the glioblastoma cells whereas SEMA3A and SEMA3B expression just caused persistent cell shape contraction [47]. On the other hand, SEMA3B was previously demonstrated to inhibit proliferation of breast and lung cancer cell lines *in vitro* [20, 22]. Some studies suggest that the mechanism of the tumor- and angio-suppressive properties of SEMA3 proteins is competition between SEMA3 and VEGF for binding to neuropilin receptors [19, 48]. Other studies reveal SEMA3 growth-inhibitory properties independent of competition with VEGF [10]. Several studies indicate that *SEMA3A* and *SEMA3F* are potent inhibitors of metastasis and angiogenesis [10, 49, 50]. Semaphorins 3 were suggested as potential angiogenesis inhibitory agents for triple negative breast cancer treatment [51]. However, Varshavsky et al. have determined that point mutations at the cleavage site of SEMA3B can inhibit angiogenesis *in vitro* and *in vivo* [52]. Joseph et al. demonstrated that forced expression of SEMA3B, but not SEMA3F, inhibited the viability of ovarian cancer cells lines *in vitro*, and presence of SEMA3F but not SEMA3B significantly inhibited the production of endothelial tubes formed by normal human umbilical vein endothelial cells (HUVEC) *in vitro* [53]. In this study, using PI-FACS we have shown that tumor growth suppression by SEMA3B is associated with induction of apoptosis *in vitro*. In addition, we have suggested that SEMA3B could be able to induce apoptosis and, possibly, inhibit angiogenesis *in vivo*, which was previously demonstrated only *in vitro* [20, 22].

One of the hallmarks of tumor-suppressor genes is the down-regulation of their expression in various tumors at the mRNA or protein level. Inactivation of suppressor genes in tumors can be caused by methylation of their promoter regions. Hypermethylation often reduces the mRNA levels not only of *SEMA3B* but also of some other genes of the LUCA region in tumors; e.g., *RASSF1A*, *BLU*, and *CACNA2D2* [36–38]. *SEMA3B* methylation has been observed in various types of cancer, including lung, liver, gallbladder, gastric, breast and oral carcinomas, and neuroblastoma [17, 54–61]. However, these studies included only the CpG-island, which, according to the NCBI database (http://www.ncbi.nlm.nih.gov/), belongs to the first intron (+1350..+1700 bp from *SEMA3B* 5’-end) of *SEMA3B*. However, there is a true promoter region, which includes CpG-dinucleotides with higher density (-300..+50 bp from *SEMA3B* 5’-end) and is capable of binding transcription factors, such as Lyf−1, DeltaEF−1, Tcf−11, c−Myb, SP1, C/EBP, and AhR/Arnt (according to the recent ENCODE data, http://genome.ucsc.edu/ENCODE/).

Here, for the first time, methylation of both intronic and promoter CpG islands has been analyzed in 14 lung and renal cancer cell lines and representative sets of primary tumors (38 NSCLC and 83 ccRCC cases). We observed frequent methylation of intronic and promoter CpG-islands in both lung (SCC and ADC) and renal primary tumors. In addition, the methylation frequency detected in the promoter CpG-island was higher than in the intronic island in SCC, ADC, and ccRCC (44–52% vs. 32–39%). Thus, methylation of both promoter and intronic *SEMA3B* CpG-islands is a feature of lung and renal tumors.

In addition, an association was observed between the decrease of the *SEMA3B* mRNA level and the methylation of the promoter and intronic CpG-islands in ccRCC primary tumors (the Spearman’s correlation coefficient was equal to 0.50 (*P* < 0.01) for the promoter CpG-island and 0.25 (*P* < 0.01) for the intronic island). These data imply a primary contribution of promoter CpG-island methylation to down-regulation of the *SEMA3B* suppressor gene.

The frequency of *SEMA3B* promoter hypermethylation (44–52%) is approximately two times lower than the frequency of expression decreases (84–95% cases, according to semi-quantitative PCR data) in NSCLC and ccRCC. Thus, other mechanisms could lead to *SEMA3B* down-regulation. For example, we have found hemi- or homozygous deletions of the 5’Sema5 marker in some ccRCC samples. There are several reports, including ours, of chromosome 3 allelic losses resulting in decreased *SEMA3B* mRNA levels in lung and renal tumors [14, 55]. The impact of non-coding RNA, especially miRNA, should not be ignored; e.g., miR-137 or miR-193a, as predicted by miRWalk (http://www.ma.uni-heidelberg.de/apps/zmf/mirwalk/).

The use of a representative set of primary tumors allowed us to reveal the correlations between the methylation frequency of both the promoter and the intronic CpG-islands of *SEMA3B* with tumor stage and grade for histological types of NSCLC (SCC and ADC) and for ccRCC. The highest Spearman’s rank correlation coefficients between mentioned features were observed for SCC (*r*_*s*_ was localized over the range 0.37–0.68).

*SEMA3B* expression was evaluated by quantitative PCR using an additional set of NSCLC (SCC and ADC) and ccRCC samples. We observed frequent and significant *SEMA3B* down-regulation in SCC and ADC, and somewhat less in ccRCC. *SEMA3B* down-regulation has been shown in lung, liver, breast, ovarian, renal and colon primary tumors using different semi-quantitative methods [2, 62]. Down-regulation of *SEMA3B* gene was negatively correlated with tumor size and gastric cancer staging [61]. Here, a large decrease of *SEMA3B* mRNA level in primary NSCLC and ccRCC was shown for the first time using qPCR. We also observed an association of the frequency and extent of the decrease in *SEMA3B* mRNA level with the development of ADC metastases (*P* < 0.05). In SCC, we revealed 10-fold and above down-regulation of the expression of this gene already at early stages of tumor development.

The methylation analysis revealed a significant association of *SEMA3B* hypermethylation with tumor progression in terms of tumor stage and grade for both subtypes of NSCLC (SCC and ADC) and ccRCC. Moreover, our qPCR expression studies have established a strong reverse correlation between the *SEMA3B* mRNA level and the presence of metastases in lung ADC. Therefore, hypermethylation of *SEMA3B* CpG-islands could be suggested as a progression marker for ccRCC and NSCLC (especially SCC), and for the *SEMA3B* mRNA level as a marker of metastasis development in lung ADC.

Other members of class 3 semaphorins also reveal distinct features, such as expression level variations or tumor suppressive ability, depending on the tumor type or the model system. Mouse melanoma (B16F10) cells overexpressing Sema3A show a significant inhibition of cell motility, invasiveness and proliferation, as well as suppression of tumor growth *in vivo*, and angiogenesis and metastasis in mice models [50]. Sema3A/B/C/E are also involved in the lymph node metastasis of prostate cancer, but they are likely to modulate the behavior of prostate cancer with a pro-tumor or anti-tumor effect, depending on the subtype [63].

In conclusion, our *in vitro* and *in vivo* results reveal the tumor suppressor role of *SEMA3B*, which could acts by inducing apoptosis or, possibly, inhibiting angiogenesis. Our data also show that methylation of both the promoter and intronic CpG-islands of *SEMA3B* is a frequent event in ccRCC and two major histological types of NSCLC, but correlation of *SEMA3B* down-regulation and hypermethylation is stronger for the promoter CpG-island. Significant decreases in the level of *SEMA3B* mRNA in the majority of the lung and renal tumor samples were revealed by qPCR. Association of *SEMA3B* hypermethylation and down-regulation with tumor progression could serve as prognostic markers. An understanding of the role of SEMA3s, including SEMA3B, in carcinogenesis will help to use their tumor suppressive and anti-angiogenic properties for the development of new agents for cancer therapy in future.

## Supporting Information

S1 Table Pathological and histological characteristics of tumors. (DOC)

S2 Table Primers, probes and PCR conditions used in the study and sizes of products. (DOC)
