# Supplementary material for: Tumor Suppressor Function of the SEMA3B Gene in Human Lung and Renal Cancers
Source: PLoS One. 2015 May 11;10(5):e0123369. doi: 10.1371/journal.pone.0123369 (PMC4427300; doi:10.1371/journal.pone.0123369)
Supplement: S1 Table — (DOC) [file pone.0123369.s001.doc]

**S1 Table.** Pathological and histological characteristics of tumors.

| **Methylation and**  **semi-quantitative RT-PCR** | | | | **qPCR** | | |
| --- | --- | --- | --- | --- | --- | --- |
| **№** | **TNM** | **Stage** | **Grade** | **№** | **TNM** | **Stage** |
| **Lung ADC** | | | | | | |
| 1 | T1N0M0 | I | 2 | 1 | T1N0M0 | I |
| 2 | T1N0M0 | I | 1 | 2 | T1N0M0 | I |
| 3 | T2N0M0 | I | 3 | 3 | T1N0M0 | I |
| 4 | T2N0M0 | I | 2 | 4 | T1N0M0 | I |
| 5 | T2N0M0 | I | 2 | 5 | T1N0M0 | I |
| 6 | T2N0M0 | I | 2 | 6 | T2N0M0 | I |
| 7 | T3N0M0 | II | 2 | 7 | T2N0M0 | I |
| 8 | T1N1M0 | II | 3 | 8 | T2N1M0 | II |
| 9 | T1N2M0 | III | 1 | 9 | T2N2M0 | III |
| 10 | T2N2M0 | III | 2 | 10 | T2N2M0 | III |
| 11 | T2N2M0 | III | 3 | 11 | T2N2M0 | III |
| 12 | T2N2M0 | III | 3 | 12 | T3N2M0 | III |
| 13 | T2N2M0 | III | 2 | 13 | T4N2M0 | III |
| 14 | T3N2M0 | III | 3 |  |  |  |
| 15 | T4N2M0 | III | 3 |  |  |  |
| 16 | T2N2M1 | IV | 3 |  |  |  |
| **Lung SCC** | | | | | | |
| 1 | T1N0M0 | I | 1 | 1 | T1N0M0 | I |
| 2 | T2N0M0 | I | 1 | 2 | T1N0M0 | I |
| 3 | T2N0M0 | I | 1 | 3 | T2N0M0 | I |
| 4 | T2N0M0 | I | 2 | 4 | T2N0M0 | I |
| 5 | T2N0M0 | I | 2 | 5 | T2N0M0 | I |
| 6 | T2N0M0 | I | 2 | 6 | T2N0M0 | I |
| 7 | T2N0M0 | I | 2 | 7 | T2N1M0 | II |
| 8 | T3N0M0 | II | 3 | 8 | T2N1M0 | II |
| 9 | T3N0M0 | II | 3 | 9 | T2N1M0 | II |
| 10 | T1N1M0 | II | 2 | 10 | T2N1M0 | II |
| 11 | T2N1M0 | II | 1 | 11 | T2N1M0 | II |
| 12 | T2N1M0 | II | 3 | 12 | T2N1M0 | II |
| 13 | T2N1M0 | II | 2 | 13 | T1N2M0 | III |
| 14 | T2N1M0 | II | 2 | 14 | T2N2M0 | III |
| 15 | T2N1M0 | II | 2 | 15 | T2N2M0 | III |
| 16 | T3N0M0 | III | 3 | 16 | T2N2M0 | III |
| 17 | T3N0M0 | III | 2 | 17 | T3N1M0 | III |
| 18 | T3N1M0 | III | 3 | 18 | T3N2M0 | III |
| 19 | T3N2M0 | III | 3 | 19 | T3N2M0 | III |
| 20 | T3N2M0 | III | 2 |  |  |  |
| 21 | T3N2M0 | III | 2 |  |  |  |
| 22 | T4N2M0 | III | 3 |  |  |  |
| **ccRCC** | | | | | | |
| 1 | T1N0M0 | I | 2 | 1 | T1N0M0 | I |
| 2 | T1N0M0 | I | 1 | 2 | T1N0M0 | I |
| 3 | T1N0M0 | I | 2 | 3 | T1N0M0 | I |
| 4 | T1N0M0 | I | 2 | 4 | T1N0M0 | I |
| 5 | T1N0M0 | I | 1 | 5 | T1N0M0 | I |
| 6 | T1N0M0 | I | 1 | 6 | T1N0M0 | I |
| 7 | T1N0M0 | I | 2 | 7 | T1N0M0 | I |
| 8 | T1N0M0 | I | 1 | 8 | T1N0M0 | I |
| 9 | T1N0M0 | I | 1 | 9 | T1N0M0 | I |
| 10 | T1N0M0 | I | 1 | 10 | T1N0M0 | I |
| 11 | T1N0M0 | I | 2 | 11 | T1N0M0 | I |
| 12 | T1N0M0 | I | 2 | 12 | T1N0M0 | I |
| 13 | T1N0M0 | I | 2 | 13 | T1N0M0 | I |
| 14 | T1N0M0 | I | 1 | 14 | T1N0M0 | I |
| 15 | T1N0M0 | I | 2 | 15 | T1N0M0 | I |
| 16 | T1N0M0 | I | 2 | 16 | T1N0M0 | I |
| 17 | T1N0M0 | I | 1 | 17 | T1N0M0 | I |
| 18 | T1N0M0 | I | 1 | 18 | T1N0M0 | I |
| 19 | T1N0M0 | I | 1-2 | 19 | T1N0M0 | I |
| 20 | T1N0M0 | I | 1 | 20 | T1N0M0 | I |
| 21 | T_1-2_N0M0 | II | 1 | 21 | T1N0M0 | I |
| 22 | T2N0M0 | II | 2 | 22 | T1N0M0 | I |
| 23 | T2N0M0 | II | 2 | 23 | T2N0M0 | II |
| 24 | T2N0M0 | II | 2 | 24 | T2N0M0 | II |
| 25 | T2N0M0 | II | 1 | 25 | T2N0M0 | II |
| 26 | T2N0M0 | II | 2 | 26 | T2N0M0 | II |
| 27 | T2N0M0 | II | 1 | 27 | T2N0M0 | II |
| 28 | T2N0M0 | II | 1 | 28 | T2N0M0 | II |
| 29 | T2N0M0 | II | 2-3 | 29 | T2N0M0 | II |
| 30 | T2N0M0 | II | 2 | 30 | T2N0M0 | II |
| 31 | T2N0M0 | II | 1 | 31 | T2N0M0 | II |
| 32 | T2N0M0 | II | 2 | 32 | T2N0M0 | II |
| 33 | T2N0M0 | II | 1 | 33 | T2N0M0 | II |
| 34 | T2N0M0 | II | 2 | 34 | T2N0M0 | II |
| 35 | T2N0M0 | II | 3 | 35 | T3N0M0 | III |
| 36 | T2N0M0 | II | 2 | 36 | T3N0M0 | III |
| 37 | T2N0M0 | II | 2 | 37 | T3N0M0 | III |
| 38 | T2N0M0 | II | 2 | 38 | T3N0M0 | III |
| 39 | T2N0M0 | II | 3 | 39 | T3N0M0 | III |
| 40 | T2N0M0 | II | 2 | 40 | T3N0M0 | III |
| 41 | T2N0M0 | II | 2 | 41 | T3N0M0 | III |
| 42 | T2N0M0 | II | 1-2 | 42 | T3N0M0 | III |
| 43 | T2N0M0 | II | 3 | 43 | T3N0M0 | III |
| 44 | T3N0M0 | III | 3 | 44 | T3N1M0 | III |
| 45 | T3N0M0 | III | 2 | 45 | T3N1M0 | III |
| 46 | T3N0M0 | III | 2 | 46 | T3N1M0 | III |
| 47 | T3N0M0 | III | 2 | 47 | T3N1M0 | III |
| 48 | T3N0M0 | III | 2 | 48 | T3N1M0 | III |
| 49 | T3N0M0 | III | 2 | 49 | T2N0M1 | III |
| 50 | T3N0M0 | III | 3 | 50 | T3N0M1 | III |
| 51 | T3N0M0 | III | 3 |  |  |  |
| 52 | T3N0M0 | III | 3 |  |  |  |
| 53 | T3N0M0 | III | 3 |  |  |  |
| 54 | T3N0M0 | III | 2 |  |  |  |
| 55 | T3N0M0 | III | 2 |  |  |  |
| 56 | T3N0M0 | III | 3 |  |  |  |
| 57 | T3N0M0 | III | 1-2 |  |  |  |
| 58 | T3N0M0 | III | 2-3 |  |  |  |
| 59 | T3N0M0 | III | 2-3 |  |  |  |
| 60 | T3N0M0 | III | 3 |  |  |  |
| 61 | T3N0M0 | III | 2-3 |  |  |  |
| 62 | T3N0M0 | III | 2 |  |  |  |
| 63 | T1N1M0 | III | 2 |  |  |  |
| 64 | T1N1M0 | III | 3 |  |  |  |
| 65 | T2N1M0 | III | 3 |  |  |  |
| 66 | T3N1M0 | III | 3 |  |  |  |
| 67 | T3N1M0 | III | 3 |  |  |  |
| 68 | T3N1M0 | III | 2 |  |  |  |
| 69 | T3N1M0 | III | 1-2 |  |  |  |
| 70 | T3N1M0 | III | 3 |  |  |  |
| 71 | T3N1M0 | III | 3 |  |  |  |
| 72 | T3N1M0 | III | 2 |  |  |  |
| 73 | T3NxM0 | III | 1 |  |  |  |
| 74 | T_1-2_N2M0 | IV | 3 |  |  |  |
| 75 | T3N2M0 | IV | 3 |  |  |  |
| 76 | T3N2M0 | IV | 3 |  |  |  |
| 77 | T3N2M0 | IV | 3 |  |  |  |
| 78 | T4N2M0 | IV | 3 |  |  |  |
| 79 | T3N0M1 | IV | 2 |  |  |  |
| 80 | T2N0M1 | IV | 2 |  |  |  |
| 81 | T3N0M1 | IV | 2 |  |  |  |
| 82 | T2N1M1 | IV | 3 |  |  |  |
| 83 | T4N2M1 | IV | 2-3 |  |  |  |
