# Supplementary material for: Tumor Suppressor Function of the SEMA3B Gene in Human Lung and Renal Cancers
Source: PLoS One. 2015 May 11;10(5):e0123369. doi: 10.1371/journal.pone.0123369 (PMC4427300; doi:10.1371/journal.pone.0123369)
Supplement: S2 Table — (DOC) [file pone.0123369.s002.doc]

**S2 Table. Primers, probes and PCR conditions used in the study and sizes of products.**

| **Marker** | **Sequences** | **T_ann_, °C/**  **Mg, mM** | **Product,**  **bp.** |
| --- | --- | --- | --- |
| Bisulfite sequencing | | | |
| *SEMA3B* | F: CCCCCACTCAATAATTCCCTTT  R: ATTTTGGGGTTGTTGGGGATT | 62/2.0 | 330 |
| MSP analysis | | | |
| *SEMA3B*-M_1^a^ | F: CCACTCCCGCCTAACTACCG  R: ATCGTTCGTCGTGTCGTAAAGT | 53.5/3.0 | 91 |
| *SEMA3B*-Um_1^a^ | F: ACTCCCACCTAACTACCA  R: TATTGTTTGTTGTGTTGTAA | 45.5/2.0 | 90 |
| *SEMA3B*-M_2^b^ | F: TGGTTAGGCGGGGTATTTTC  R: TCAACAATAAAAACGAAAACG | 58/3.0 | 133 |
| *SEMA3B*-Um_2^b^ | F: GTGGTTAGGTGGGGTATTTTT  R: ATCAACAATAAAAACAAAAACA | 58/3.0 | 135 |
| Semi-quantitative RT-PCR | | | |
| *MHCI* | F: GCAAGGATTACATCGCCCTGAACGAG  R: CATCATAGCGGTGACCACAGCTCCAA | 60/2.5 | 1276^c^ |
| *SEMA3B* | F: TTCTTTCGTGAGACGGCGGTA  R: CCCTGGAAGATGCTGCTGGA | 58/2.5 | 275 |
| *B2M* | F: TGACTTTGTCACAGCCCAAGATAG  R: CAAATGCGGCATCTTCAAACCTC | 64/2.5 | 81 |
| qPCR | | | |
| *SEMA3B* | F: CCTCAACCTGGACAACATCAGC  R: CGAAGTTCATGCACTCAGTACCAAT  Pr: CCAGTTGCACTCCTCTCGCCATTCC | 60/2.5 | 119 |
| *GAPDH* | F: CGGAGTCAACGGATTTGGTC  R: TGGGTGGAATCATATTGGAACAT  Pr: CCTTCATTGACCTCAACTACATGGTTTACAT | 60/2.5 | 141 |
| *GUSB* | F: GATGGAAGAAGTGGTGCGTAGG  R: TTAGAGTTGCTCACAAAGGTCACAG  Pr: CGTCCCACCTAGAATCTGCTGGCTACTACTT | 60/2.5 | 171 |
| *RPN1* | F: CACCCTCAACAGTGGCAAGAAG  R: TGCATTTCGCTCACTCTGTCG  Pr: CCCTCTGTCTTCAGCCTGGACTGCA | 60/2.5 | 126 |

***Note:*** T_ann_ **–** PCR annealing temperature; F – forward primer; R – reverse primer; Pr – probe. **a – used in the analysis of the 1-st (promoter) CpG-island; b – used in the analysis of the 2-nd (intronic) CpG-island ; c – test for DNA contaminated cDNA (the product size for *MHCI* from cDNA is about 300 bp.). All primers were designed here besides mentioned .**

**References**

1. Kuroki T, Trapasso F, Yendamuri S, Matsuyama A, Alder H, Williams NN, et al. Allelic loss on chromosome 3p21.3 and promoter hypermethylation of semaphorin 3B in non-small cell lung cancer. Cancer research. 2003;63(12): 3352-5.

2. Angeloni D, ter Elst A, Wei MH, van der Veen AY, Braga EA, Klimov EA, et al. Analysis of a new homozygous deletion in the tumor suppressor region at 3p12.3 reveals two novel intronic noncoding RNA genes. Genes, chromosomes & cancer. 2006;45(7): 676-91.
